# A Note on Parametric Surfaces in Minkowski 3-Space

**DOI:** 10.1155/2014/618340

**Published:** 2014-07-10

**Authors:** Esra Betul Koc Ozturk

**Affiliations:** Department of Mathematics, Faculty of Sciences, University of Çankiri Karatekin, 18100 Çankiri, Turkey

## Abstract

With the help of the Frenet frame of a given pseudo null curve, a family of parametric surfaces is expressed as a linear combination of this frame. The necessary and sufficient conditions are examined for that curve to be an isoparametric and asymptotic on the parametric surface. It is shown that there is not any cylindrical and developable ruled surface as a parametric surface. Also, some interesting examples are illustrated about these surfaces.

## 1. Introduction

The Minkowski 3-space *E*_1_^3^ is the Euclidean 3-space *E* ^3^ provided with the standard flat metric given by

(1) $$\begin{array}{c} g=-dx_{1}^{2}+dx_{2}^{2}+dx_{3}^{2}, \end{array}$$

where (*x*_1_, *x*_2_, *x*_3_) is a rectangular coordinate system of *E*_1_^3^. Since *g* is an indefinite metric, recall that a vector *v* ∈ *E*_1_^3^ can have one of three Lorentzian causal characters: it can be spacelike if *g*(*v*, *v*) > 0 or *v* = 0, timelike if *g*(*v*, *v*) < 0, and null (lightlike) if *g*(*v*, *v*) = 0 and *v* ≠ 0. In particular, the norm (length) of a vector *v* is given by $||v||=\sqrt{\left|g(v,v)\right|}$ and two vectors $\vec{v}$ and $\vec{w}$ are said to be orthogonal, if *g*(*v*, *w*) = 0. Next, recall that an arbitrary curve *α* = *α*(*s*), in *E*_1_^3^, can locally be* spacelike*,* timelike*, or* null (lightlike)*, if all of its velocity vectors *α*′(*s*) are, respectively,* spacelike*,* timelike*, or* null (lightlike)* [1]. In Minkowski 3-space, a spacelike curve whose principal normal *N* and binormal *B* are null vectors is called* pseudo null curve* [2].

If *α* is a pseudo null curve, the Frenet formulas have the form [2, 3]

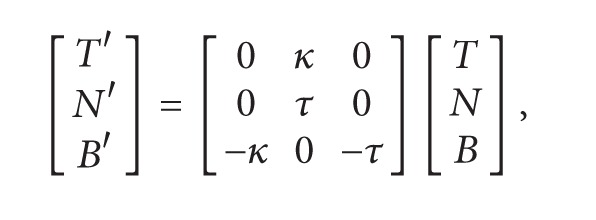
(2)
where

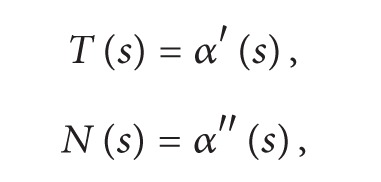
(3)

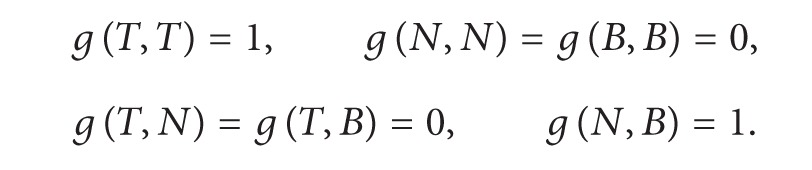
(4)
For a pseudo null curve, the first curvature *κ* can take only two values: *κ* = 0 when *α* is a straight line, or *κ* = 1 in all other cases [2, 3].

Also, we have

(5) $$\begin{array}{c} T\times N=N,\;\;N\times B=T,\;\;B\times T=B. \end{array}$$

In the differential geometry of surfaces, an asymptotic curve is formally defined as a curve on a regular surface such that the normal curvature is zero in the asymptotic direction. Asymptotic directions can only occur when the Gaussian curvature on surface is negative or zero along the asymptotic curve [1, 4, 5].

Asymptotic curves or asymptotics have been the subject in differential geometry, in architectural CAD, and in molecular design (see [6]). There are recent works about asymptotics: Wang et al. [7] introduced the concept of surface pencil with a common isogeodesic curve. Bayram et al. [8] obtained the parametric representation for a surface pencil from a given curve as an isoparametric and asymptotic curve in Minkowski 3-space *E*_1_^3^. Abdel-Baky and Al-Ghefari in [9] demonstrated some interesting ruled and developable surfaces as a surface pencil from a given asymptotic curve. Also, Saffak et al. [10] expressed a family of surfaces from a given spacelike or timelike asymptotic curve using the Frenet trihedron frame of the curve in Minkowski 3-space *E*_1_^3^.

The goal of the study is to construct the parametric representation of surface from a given pseudo null curve and derive the necessary and sufficient conditions for the given pseudo null curve to be an isoparametric and asymptotic on the parametric surface. The family of parametric surfaces with common pseudo null asymptotic curve is defined. Also, it is shown that there is not any cylindrical and developable ruled surface as a parametric surface and some interesting examples about these surfaces are illustrated.

In this paper we will assume that pseudo null base curve *α* has the first curvature *κ*(*s*) = 1; that is, that the curve *α* is not a straight line.

## 2. Surfaces with Common Asymptotic Curve in *E*_1_^3^

Let *S* be a parametric surface on a pseudo null curve *α* = *α*(*s*) in the 3-dimensional Minkowski space with parametrization

(6) $$\begin{array}{c} \phi :\left[0,L\right]\times \left[0,T\right]⟶E_{1}^{3},\\ \left(s,t\right)⟶\phi \left(s,t\right)=\alpha \left(s\right)+e\left(s,t\right), \end{array}$$

where *a*(*s*, *t*),  *b*(*s*, *t*), and *c*(*s*, *t*) are *C* ^1^ functions and **e** is given by

(7) $$\begin{array}{c} e\left(s,t\right)=a\left(s,t\right)T\left(s\right)+b\left(s,t\right)N\left(s\right)+c\left(s,t\right)B\left(s\right). \end{array}$$

If the parameter *t* is seen as the time, the functions *a*(*s*, *t*), *b*(*s*, *t*), and *c*(*s*, *t*) can then be viewed as directed marching distances of a point unit in the time *t* in the direction *T*, *N*, and *B*, respectively, and the position vector *α* is seen as the initial location of this point.

By taking the derivative of (7) with respect to *s* and using the Frenet equations (2), we get

(8) es=∂e∂s=(as−c)T+(bs+bτ+a)N+(cs−cτ)B.

The normal *η*(*s*, *t*) of the surface *S* is given by

(9) $$\begin{array}{c} \eta \left(s,t\right)=\phi_{s}\times \phi_{t}, \end{array}$$

and since

(10) $$\begin{array}{c} \phi_{s}=\frac{\partial \phi }{\partial s}=\left(a_{s}-c+1\right)T+\left(b_{s}+b\tau +a\right)N+\left(c_{s}-c\tau \right)B,\\ \phi_{t}=\frac{\partial \phi }{\partial t}=a_{t}T+b_{t}N+c_{t}B, \end{array}$$

using (5), the normal vector can be written as

(11) η(s,t)=((bs+bτ+a)ct−(cs−cτ)bt)T +((as−c+1)bt−(bs+bτ+a)at)N +((cs−cτ)at−(as−c+1)ct)B.

Let the curve *α* = *α*(*s*) on the ruled surface *S*, given by (6), be an isoparametric. Then there should exist a parameter *t* = *t*_0_ such that *α*(*s*) = *ϕ*(*s*, *t*_0_) where

(12) $$\begin{array}{c} a\left(s,t_{0}\right)=b\left(s,t_{0}\right)=c\left(s,t_{0}\right)=0. \end{array}$$

And from (11) we obtain

(13) η(s,t0)=(bsct−csbt)T+((as+1)bt−bsat)N +(csat−(as+1)ct)B.

According to [11], the curve *α* on the surface *S* is asymptotic if and only if the binormal *B*(*s*) of the curve *α* and the normal *η*(*s*, *t*_0_) of the surface *S* at any point on the curve *α* are parallel to each other. Thus for all *s* ∈ [0, *L*]

(14) $$\begin{array}{c} B\left(s\right)||\eta \left(s,t_{0}\right) \end{array}$$

if and only if

(15) $$\begin{array}{c} b_{s}c_{t}-c_{s}b_{t}=0,\\ \left(a_{s}+1\right)b_{t}-b_{s}a_{t}=0,\\ c_{s}a_{t}-\left(a_{s}+1\right)c_{t}\neq 0. \end{array}$$

Therefore, we can give the necessary and sufficient conditions for the surface *S* to have the pseudo null curve *α* as an isoparametric and asymptotic with the following theorem.

**Theorem 1 .**

Let *S* be a surface having a pseudo null base curve *α* in the 3-dimensional Minkowski space with parametrization (6). The curve *α* is an isoparametric and asymptotic curve on the surface *S* if and only if the following conditions are satisfied:

(16) $$\begin{array}{c} a\left(s,t_{0}\right)=b\left(s,t_{0}\right)=c\left(s,t_{0}\right)=0,\\ b_{s}c_{t}-c_{s}b_{t}=0,\\ \left(a_{s}+1\right)b_{t}-b_{s}a_{t}=0,\\ c_{s}a_{t}-\left(a_{s}+1\right)c_{t}\neq 0, \end{array}$$

where *a*(*s*, *t*), *b*(*s*, *t*), and *c*(*s*, *t*) are *C* ^1^ functions.

We call the set of surfaces defined by (6) and (15)* the family of surfaces with common isoasymptotic*, since the common isoasymptotic is also an isoparametric curve on these surfaces. Any surface *S* defined by (6) and satisfying conditions (12) and (15) is a member of the family.

As mentioned in [7], the marching-scale functions *a*(*s*, *t*), *b*(*s*, *t*), and *c*(*s*, *t*) can be decomposed into two factors.

**Case 1 .**

If we choose

(17) a(s,t)=l(s)A(t),b(s,t)=m(s)B(t),c(s,t)=n(s)C(t),

where *l*(*s*), *m*(*s*), *n*(*s*), *A*(*t*), *B*(*t*), and *C*(*t*) are *C* ^1^ functions, and *l*(*s*), *m*(*s*), and *n*(*s*) are not identically zero, then, from Theorem 1, we can simply express that the necessary and sufficient condition of the curve *α* being an isoparametric and asymptotic curve on the surface *S* is

(18) $$\begin{array}{c} A\left(t_{0}\right)=B\left(t_{0}\right)=C\left(t_{0}\right)=0,\\ \frac{dB\left(t_{0}\right)}{dt}=0,\;\;m\left(s\right)=0,\\ \frac{dC\left(t_{0}\right)}{dt}\text{const}\neq 0,\;\;n\left(s\right)\neq 0. \end{array}$$

For the case when the marching-scale functions *a*(*s*, *t*),  *b*(*s*, *t*), and *c*(*s*, *t*) depend only on the parameter *t*, if we choose *l*(*s*) = *m*(*s*) = *n*(*s*) = 1, then the corresponding family of surfaces with the common isoasymptotic becomes

(19) $$\begin{array}{c} \phi \left(s,t\right)=\alpha \left(s\right)+A\left(t\right)T\left(s\right)+B\left(t\right)N\left(s\right)+C\left(t\right)B\left(s\right). \end{array}$$

By simplifying, condition (18) can be represented as

(20) $$\begin{array}{c} A\left(t_{0}\right)=B\left(t_{0}\right)=C\left(t_{0}\right)=0,\\ \frac{dB\left(t_{0}\right)}{dt}=0,\;\;\frac{dC\left(t_{0}\right)}{dt}\text{const}\neq 0. \end{array}$$

**Case 2 .**

If we choose

(21) a(s,t)=f(l(s)A(t)),b(s,t)=g(m(s)B(t)),c(s,t)=h(n(s)C(t)),

where *l*(*s*), *m*(*s*), *n*(*s*), *A*(*t*), *B*(*t*), *C*(*t*), *f*, *g*, and *h* are *C* ^1^ functions, then, from Theorem 1 and (18), we can simply express that the necessary and sufficient condition of the curve *α* being an isoparametric and asymptotic curve on the surface *S* is

(22) $$\begin{array}{c} A\left(t_{0}\right)=B\left(t_{0}\right)=C\left(t_{0}\right)=0,\\ f\left(0\right)=g\left(0\right)=h\left(0\right)=0,\\ \frac{dB\left(t_{0}\right)}{dt}=0,\;\;m\left(s\right)=0,\;\;g^{\prime }\left(0\right)=0,\\ \frac{dC\left(t_{0}\right)}{dt}\text{const}\neq 0,\;\;n\left(s\right)\neq 0,\;\;h^{\prime }\left(0\right)\neq 0. \end{array}$$

The choices given above give an advantage: any set of functions *l*(*s*), *m*(*s*), and *n*(*s*) would satisfy (18) or (22). Thus we can select different sets of functions *l*(*s*), *m*(*s*), and *n*(*s*) to adjust the shape of the surface until they are gratified with the design, and the resulting surface is guaranteed to belong to the surface family with the pseudo null curve *α*(*s*) as the common asymptotic.

**Example 2 .**

Let *S* be a parametric surface on a pseudo null curve *α* = *α*(*s*) in the 3-dimensional Minkowski space parameterized by

(23) $$\begin{array}{c} \phi :\left[0,L\right]\times \left[0,T\right]⟶E_{1}^{3},\\ \left(s,t\right)⟶\phi \left(s,t\right)=\alpha \left(s\right)+e\left(s,t\right), \end{array}$$

where **e** satisfy the conditions (7).

**Figure 1:**
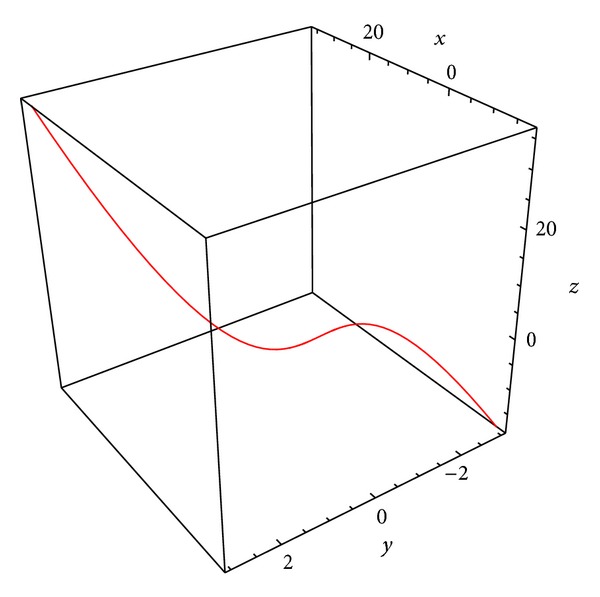
The curve *α*.

As a curve *α*, consider the pseudo null curve (see Figure 1)

(24) $$\begin{array}{c} \alpha \left(s\right)=\left(s^{3}+s^{2},s,s^{3}+s^{2}\right). \end{array}$$

Then we get the Frenet vectors as follows:

(25) $$\begin{array}{c} \vec{T}\left(s\right)=\alpha^{\prime }\left(s\right)=\left(3s^{2}+2s,1,3s^{2}+2s\right),\\ \vec{N}\left(s\right)=\alpha^{\prime \prime }\left(s\right)=\left(6s+2,0,6s+2\right),\\ \vec{B}\left(s\right)=\left(-\frac{1+{\left(3s^{2}+2s\right)}^{2}}{2},-\left(3s^{2}+2s\right),\frac{1-{\left(3s^{2}+2s\right)}^{2}}{2}\right). \end{array}$$

Moreover, the curvatures *κ* and *τ* of *α* have the form

(26) $$\begin{array}{c} \kappa \left(s\right)=1,\;\;\tau \left(s\right)=\frac{6}{6s+2}. \end{array}$$

If we choose

(27) a(s,t)=γs,b(s,t)=0,c(s,t)=δs2,

where *γ*, *δ* ∈ *R*, *δ* ≠ 0, then the surfaces family with the common isoasymptotic is given by

(28) $$\begin{array}{c} \phi \left(s,t\right)=\left(\begin{array}{c} -\frac{1}{2}s^{2}\left(t\delta -4t\gamma -2s-6st\gamma +4s^{2}t\delta +12s^{3}t\delta +9s^{4}t\delta -2\right),\\ s\left(-3t\delta s^{3}-2t\delta s^{2}+t\gamma +1\right),\\ \frac{1}{2}s^{2}\left(2s+4t\gamma +t\delta +6st\gamma -4s^{2}t\delta -12s^{3}t\delta -9s^{4}t\delta +2\right) \end{array}\right). \end{array}$$

**Figure 2:**
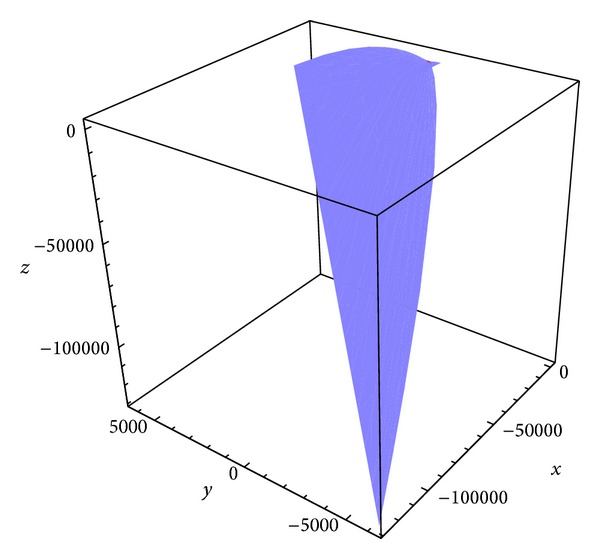
A member of surface family for *γ* = 1 and *δ* = 1 and the curve *α*.

For *γ* = 1 and *δ* = 1 we obtain a member of the surface (see Figure 2) as

(29) $$\begin{array}{c} \phi \left(s,t\right)=\left(\begin{array}{c} \frac{1}{2}s^{2}\left(2s+3t-4s^{2}t-12s^{3}t-9s^{4}t+6st+2\right),\\ s\left(-3ts^{3}-2ts^{2}+t+1\right),\\ \frac{1}{2}s^{2}\left(2s+5t-4s^{2}t-12s^{3}t-9s^{4}t+6st+2\right) \end{array}\right), \end{array}$$

where *s* ∈ (−3,5] and *t* ∈ [−5,5].

**Figure 3:**
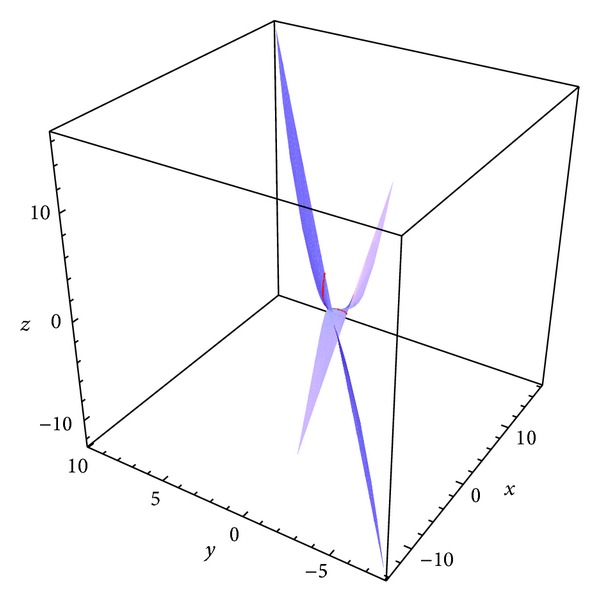
A member of surface family for *γ* = 1 and *δ* = cosh⁡*s* and the curve *α*.

For *γ* = 1 and *δ* = cosh⁡*s* we obtain a member of the surface (see Figure 3) as

(30) $$\begin{array}{c} \phi \left(s,t\right)=\left(\begin{array}{c} 2s^{2}t+3s^{3}t+s^{2}+s^{3}+\frac{1}{2}s^{2}t\cosh s+2s^{4}t\cosh s+6s^{5}t\cosh s+\frac{9}{2}s^{6}t\cosh s,\\ s+st+2s^{3}t\cosh s+3s^{4}t\cosh s,\\ 2s^{2}t+3s^{3}t+s^{2}+s^{3}-\frac{1}{2}s^{2}t\cosh s+2s^{4}t\cosh s+6s^{5}t\cosh s+\frac{9}{2}s^{6}t\cosh s \end{array}\right), \end{array}$$

where *s* ∈ (−3,5] and *t* ∈ [−5,5].

## 3. Ruled Surface with Common Asymptotic Curve

Let *α* = *α*(*s*) be a pseudo null curve in the 3-dimensional Minkowski space. Suppose *ϕ*(*s*, *t*) is a ruled surface with the directrix *α*(*s*) which is also an isoparametric curve of *ϕ*(*s*, *t*). In that case, there exists a parameter *t* = *t*_0_ such that *ϕ*(*s*, *t*_0_) = *α*(*s*) for all *s* ∈ [0, *L*], then for *s* ∈ [0, *L*], *t* ∈ [0, *T*], and *t*_0_ ∈ [0, *L*] the surface *ϕ*(*s*, *t*) can be expressed as

(31) $$\begin{array}{c} \phi \left(s,t\right)-\phi \left(s,t_{0}\right)=\left(t-t_{0}\right)D\left(s\right), \end{array}$$

where *D*(*s*) denotes the direction of the rulings.

Also, from (6) and (7), we get

(32) $$\begin{array}{c} \left(t-t_{0}\right)D\left(s\right)=a\left(s,t\right)T\left(s\right)+b\left(s,t\right)N\left(s\right)+c\left(s,t\right)B\left(s\right) \end{array}$$

and we get a system of three equations with three unknown functions *a*(*s*, *t*), *b*(*s*, *t*), and *c*(*s*, *t*) as follows:

(33) a(s,t)=(t−t0)det⁡[D(s),N(s),B(s)],b(s,t)=(t−t0)det⁡[D(s),B(s),T(s)],c(s,t)=(t−t0)det⁡[D(s),T(s),N(s)].

The above equations in (33) are just the necessary and sufficient conditions for which *ϕ*(*s*, *t*) is a ruled surface with a directrix *α*(*s*).

If the curve *α*(*s*) is also asymptotic on the surface *ϕ*(*s*, *t*), by using the conditions given in (18), then for all *s* ∈ [0, *L*] these conditions become

(34) det⁡[D(s),B(s),T(s)]=0,det⁡[D(s),T(s),N(s)]≠0.

It follows that, at any point on the curve *α*(*s*), the ruling direction *D*(*s*) must be in the plane formed by *T*(*s*) and *B*(*s*). Moreover, the ruling direction *D*(*s*) and the vector *T*(*s*) must not be parallel. Thus, for some real functions *u*(*s*) and *v*(*s*), we can write

(35) $$\begin{array}{c} D\left(s\right)=u\left(s\right)T\left(s\right)+v\left(s\right)B\left(s\right), \end{array}$$

where *v*(*s*) ≠ 0 for all *s* ∈ [0, *L*]. Substituting it into the expressions in (33)–(35), we have

(36) a(s,t)=tu(s),b(s,t)=0,c(s,t)=tv(s),

where *c*(*s*, *t*) ≠ 0 for all *s* ∈ [0, *L*].

Thus, the isoasymptotic ruled surface with the common asymptotic directrix *α*(*s*) is given by

(37) $$\begin{array}{c} \phi \left(s,t\right)=\alpha \left(s\right)+t\left(u\left(s\right)T\left(s\right)+v\left(s\right)B\left(s\right)\right), \end{array}$$

where the real functions *u*(*s*) and *v*(*s*) control the shape of the ruled surface, and *v*(*s*) ≠ 0 for all *s* ∈ [0, *L*]. On the other hand, there exist two asymptotic curves passing through every point on the curve *α*(*s*): one is *α*(*s*) itself and the other is a straight line in the direction *D*(*s*) as given in (35). Every member of the isoasymptotic ruled surface is decided by two parameters *u*(*s*) and *v*(*s*), that is, by the direction vector function *D*(*s*).

From (4) and (35), for all *s* ∈ [0, *L*], we have

(38) $$\begin{array}{c} g\left(D\left(s\right),D\left(s\right)\right)=u^{2}\left(s\right), \end{array}$$

and we can give the following cases.

**Case 3 .**

If *g*(*D*(*s*), *D*(*s*)) = 0 for all *s* ∈ [0, *L*], then *u*(*s*) = 0 and the direction *D*(*s*) is a null vector given by

(39) $$\begin{array}{c} D\left(s\right)=v\left(s\right)B\left(s\right), \end{array}$$

where *v*(*s*) ≠ 0 for all *s* ∈ [0, *L*]. By taking the derivative of (39) with respect to *s* and using (2), we get

(40) $$\begin{array}{c} D\left(s\right)\times D^{\prime }\left(s\right)=-v\left(s\right)T\left(s\right)+\left(v^{\prime }\left(s\right)-v\left(s\right)\tau \left(s\right)\right)B\left(s\right) \end{array}$$

and *D*(*s*) × *D*′(*s*) does not equal zero vectors because of *v*(*s*) ≠ 0 for all *s* ∈ [0, *L*]. Thus, there is not any cylindrical ruled surface as defined by (37).

Also, from (4) and (40), we have

(41) $$\begin{array}{c} g\left(D\left(s\right)\times D^{\prime }\left(s\right),\alpha^{\prime }\left(s\right)\right)=-v\left(s\right) \end{array}$$

and since *v*(*s*) ≠ 0 for all *s* ∈ [0, *L*], the ruled surface is not developable.

**Case 4 .**

If *g*(*D*(*s*), *D*(*s*)) ≠ 0 for all *s* ∈ [0, *L*], then *u*(*s*) ≠ 0 and the direction *D*(*s*) is a spacelike vector. By taking the derivative of (35) with respect to *s* and using (2), we get

(42) D(s)×D′(s)=−u(s)v(s)T(s)+u2(s)N(s) +(v(s)u′(s)−u(s)v′(s)−v2(s)+u(s)v(s)τ(s))B(s)

and this equation does not equal zero vectors because of *u*(*s*) ≠ 0 and *v*(*s*) ≠ 0 for all *s* ∈ [0, *L*]. Thus, there is not any cylindrical ruled surface as defined by (37).

Also, from (4)-(5) and (35), we have

(43) $$\begin{array}{c} g\left(D\left(s\right)\times D^{\prime }\left(s\right),\alpha^{\prime }\left(s\right)\right)=-u\left(s\right)v\left(s\right) \end{array}$$

and since *u*(*s*) ≠ 0 and *v*(*s*) ≠ 0 for all *s* ∈ [0, *L*], the ruled surface is not developable.

Therefore, from Cases 3 and 4, we can give the following corollary.

**Corollary 3 .**

There is not any cylindrical and developable ruled surface as defined by  (37).

**Example 4 .**

Let *S* be a ruled surface whose asymptotic curve is the pseudo null curve in Example 2.

**Figure 4:**
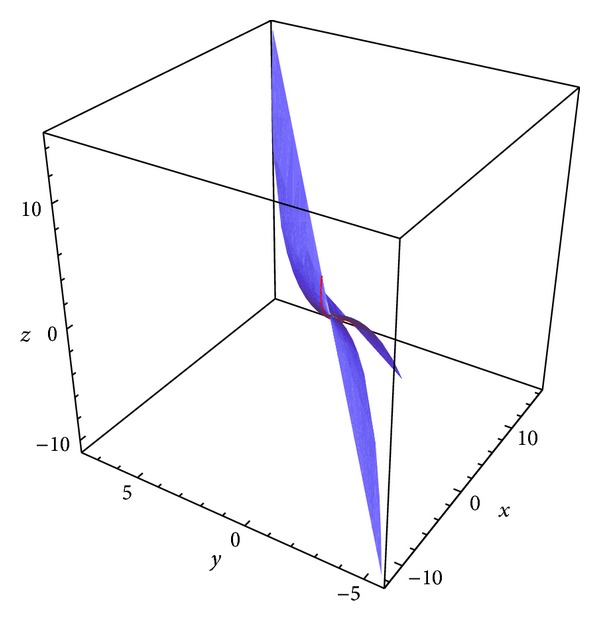
The cylindrical ruled surface *φ* with pseudo null curve.

If the controlling functions of the ruled surface are

(44) $$\begin{array}{c} u\left(s\right)=\sinh\;s,\\ v\left(s\right)=\cosh\;s, \end{array}$$

then the corresponding cylindrical surface is shown in Figure 4.

**Figure 5:**
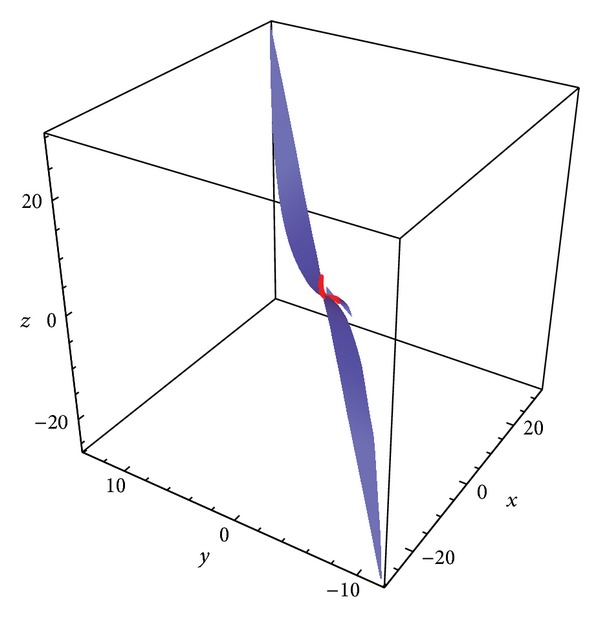
The non-cylindrical ruled surface *φ* with pseudo null curve.

If the controlling functions of the ruled surface for all *s* are

(45) $$\begin{array}{c} u\left(s\right)=s,\\ v\left(s\right)=e^{s}, \end{array}$$

then the corresponding noncylindrical surface is shown in Figure 5.
